# Estimating Potential for Drug Budget Reallocation Following Expiration of Exclusivity of Pharmaceutical Products

**DOI:** 10.36469/jheor.2022.29624

**Published:** 2022-02-03

**Authors:** Samira Toghanian, John Moshtaghi-Svensson, Maria Papageorgiou, Kristin Kittelsen, Christiaan Dolk, Markus Hultstrand, Stina Salomonsson

**Affiliations:** 1 MSD, Center for Observational and Real World Evidence, Stockholm, Sweden; 2 Godjy AB, Stockholm, Sweden; 3 MSD, Athens, Greece; 4 MSD, Oslo, Norway; 5 MSD, Haarlem, The Netherlands; 6 MSD, Stockholm, Sweden

**Keywords:** forecast model, drug budget, health policy, generic, biosimilar, innovative medicine

## Abstract

**Background:** The prioritization of public funds in an equitable and ethically sound manner along with efficient budget allocation are key challenges for governments and budget holders. Following the introduction of generics/biosimilars, the potential total budget made available for reallocation resulting from the loss of exclusivity (LOE) in a given market has not been estimated.

**Objectives:** This study investigated the impact of generic/biosimilar entry on drug budget in 4 countries.

**Methods:** Pharmaceutical sales data, drug costs and LOE dates were modeled and forecast using an analytical framework (Affordability by ReallocaTing Funds model [ART]) to estimate future incremental budget availability using scenario analyses in Greece (GR), the Netherlands (NL), Norway (NO) and Sweden (SW).

**Results:** During 2020-2022, 166 (GR), 222 (NL), 145 (NO) and 93 (SW) products facing LOE were identified. This equated to release of an estimated cumulative budget during 2020-2024 of €218 million (GR), €1319 million (NL), €340 million (NO) and €876 million (SW). The estimated average budget released per year during 2020-2024 was 1.8% (GR), 4.6% (NL), 3.4% (NO) and 3.9% (SW) of each country’s total annual drug budget.

**Discussion:** These analyses showed that LOE for pharmaceutical products between 2020 and 2022 can result in significant increase in budget availability. LOE in the retail channel was the main driver of budget availability in GR and SW, compared to LOE in the hospital channel in the NL and NO.

**Conclusion:** Estimation of future release of budget capacity using the Affordability by ReallocaTing Funds model supports discussion on resource allocation to fund innovation and may help inform policy changes.

## BACKGROUND

A key challenge for healthcare decision makers around the globe is managing healthcare budgets in an environment of increasing economic pressure. Payers are tasked with ensuring the availability of new treatments to improve public health, despite only minor year-on-year increases in drug budgets.^1–4^ Prioritization of investments with public funds requires both equity and ethically sound decisions, and efficient budget allocation remains a challenge for governments and budget holders. Although the introduction of innovative medicines is considered to have put pressure on healthcare budgets,^5^ drug budgets have remained largely stable in recent years.^6,7^ This is despite the increasing healthcare needs of aging populations with chronic conditions and comorbidities driving healthcare costs (as a result of better health care) and the increasing prevalence of chronic diseases and opportunities to treat previously fatal conditions.^1,3,4,8^ Reports have shown that slight increases in pharmaceutical spending have been primarily driven by increases in volume rather than price.^9,10^ Indeed, the general trend is for drug prices to fall and market dynamics to change following loss of exclusivity (LOE) and the entrance of generics or biosimilars–factors that may not be considered in these discussions. In many countries, the price of generics and biosimilars is mandatorily set at a certain percentage below the original drug price, and the branded drug price will also decrease due to local drug pricing legislation and/or competition.^8,11^ Generics may be discounted more than 80% relative to originators, though generic pricing policies differ between countries.^1,12^ Biosimilars have higher development and manufacturing costs than generics and additional challenges with ensuring uptake. Although biosimilars are generally not discounted to the same extent as generics, they can still provide additional competitive and regulatory pricing incentives.^11,12^ As a result, less funding is required to treat the same population with the LOE product or its generics/biosimilars, opening up discussion for the reallocation of drug budget. While patent protection and other policies enabling product exclusivity are crucial to drive innovation and ensure continued investment in drug research to improve health, when this period of exclusivity ends, generics and biosimilars can play a key role in releasing funds to be reallocated elsewhere.

Although these market dynamics post-LOE are acknowledged, studies on the impact of patent expiry on the total pharmaceutical and healthcare costs, particularly in European markets, are lacking.^2^ Several budget impact and cost-effectiveness models have considered off-patent changes to prices and market share on an individual product level^13–15^; however, the potential total budget made available for reallocation as a result of all known products facing LOE in a given market has not been estimated, to our knowledge. Vataire et al (2014) modeled pharmaceutical expenditure based on the net effect of products going off-patent and newly branded medicinal products over 5 years. However, the authors explored the total forecasted change in budget rather than assessing the potential funds available as a result of LOE.^16^

The Affordability by ReallocaTing funds (ART) model was designed as a structured analytical framework to estimate the potential budget made available for reallocation as a result of LOE, to address the affordability challenge and “ability to pay” concerns of decision makers, policymakers, and payers and facilitate efficient budgeting. The study aimed to conduct scenario analyses for four countries: Sweden, Norway, the Netherlands, and Greece. These countries were selected based on their differing trends in drug list prices over time.^17^ For example, list prices in Greece and Norway have generally increased, but list prices in Sweden and the Netherlands have generally decreased.^17^ Therefore, these countries were selected to see if there were any differences by investigating these two trends using the ART model.

## METHODS

### Model Development

The objective of the ART model was to assess products expected to lose exclusivity within a specific time interval, with LOE dates estimated based on assessment of the protection status of products within a country, including country-specific patents, patent extensions, Supplementary Protection Certificates/Certificate of Pharmaceutical Products, data exclusivity, pediatric exclusivity, and ongoing litigation. The estimated LOE dates included all product-level extensions added to the underlying patent record. The model then enabled calculation of the budget made available for reallocation as a result of these LOE products, using MIDAS® data (IQVIA) for their annual sales value (moving annual total, MAT). The future budget available for reallocation was calculated by estimating the total expenditure if the LOE products identified were to maintain exclusivity throughout the analysis period and comparing this with the estimated budget spending when these products lose exclusivity. The model was developed in R with an R Shiny interface.^18^

The model calculated the potential budget available for reallocation from a single product or group of products, or to identify all LOE products based on a set of scenario inputs, which could be filtered by LOE period, sales channel (hospital/retail/combined), and Anatomical Therapeutic Chemical (ATC) code. ATC categories, as defined by the World Health Organization, are listed in **Supplementary Table 1**.^19^

Budgets available for reallocation were calculated over 6 cycles, each corresponding to 6 months, with further cycles (cycles 7-10) using inputs from cycle 6. The ART model calculated the potential budget made available for each cycle and the cumulative value based on the additional LOE products each year **(Supplementary Figure 1).** It was assumed that upon entry, generics and biosimilars absorb a share of the market at a lower price point, thus generating budget availability for reallocation. Meanwhile, it was assumed that price decreases in originator drugs, based on mandated decreases and/or additional competition, will also increase the budget available for reallocation. The forecast estimates therefore considered an assumed market penetration of generics and biosimilars, market expansion, and mandated or expected shifts in prices for both branded originator products and generics/biosimilars.

### Scenario Analyses

Scenario analyses were conducted for Greece, the Netherlands, Norway, and Sweden, including products losing exclusivity during the 3-year period from 2020-2022, with a forecast of the resulting budget available to 2024. For each country, the analysis spanned all therapeutic areas and included drugs utilized in both hospital and retail sectors.

All four countries have primarily publicly funded healthcare systems, with the government bearing the cost of most pharmaceuticals. Although similarities between the countries’ healthcare systems allowed for the same modeling approach, a set of model inputs were applied for each country to reflect the local setting with regard to expected changes in the market dynamics post-LOE **(Table 1).** Because funding systems, regulatory controls, and utilization of generics and biosimilars vary by country, direct country-to-country comparisons may not be appropriate. In addition, different inputs were used for hospital and retail sectors, as pharmaceuticals are funded through different routes across these channels, with different procurement processes and regulations on pricing and uptake post-LOE.^20–24^ The separate analysis of hospital and retail sectors also facilitates consideration of the different budget implications given the different financing routes.

**Table 1.** Input Parameters for Scenario Analyses in Greece, the Netherlands, Norway, and Sweden

**Variables**	**Input Parameters during a 3-Year Period (6 Cycles) Post-LOE (%)^a^**					
	**Cycle 1**	**Cycle 2**	**Cycle 3**	**Cycle 4**	**Cycle 5**	**Cycle 6**
**Greece**						
**Hospital**						
Market share of generics/biosimilar (%)	20	40	50	60	70	70
Market growth/expansion post-LOE (%)	0	0	0	0	0	0
Post-LOE expected price level of branded product^b^	100	93	93	87	87	80
Post-LOE expected price level of generics/biosimilars^b^	65	61	61	56	56	52
**Retail**						
Market share of generics/biosimilar (%)	10	20	40	50	60	60
Market growth/expansion post-LOE (%)	0	0	0	0	0	0
Post-LOE expected price level of branded product^b^	65	61	61	56	56	52
Post-LOE expected price level of generics/biosimilars^b^	65	61	61	56	56	52
**The Netherlands**						
**Biological products (hospital or retail)^c^**						
Market share of generics/biosimilar (%)	50	50	70	70	80	80
Market growth/expansion post-LOE (%)	0	0%	0	0	0	0
Post-LOE expected price level of branded product^b^	70	70	50	50	40	40
Post-LOE expected price level of generics/biosimilars^b^	50	50	30	30	30	30
**Small molecules (hospital or retail)^c^**						
Market share of generics/biosimilar (%)	50	50	70	70	80	80
Market growth/expansion post-LOE (%)	0	0	0	0	0	0
Post-LOE expected price level of branded product^b^	50	50	40	40	40	40
Post-LOE expected price level of generics/biosimilars^b^	30	30	20	20	20	20
**Norway**						
**Hospital**						
Market share of generics/biosimilar (%)	60	70	80	90	90	90
Market growth/expansion post-LOE (%)	0	0	0	0	0	0
Post-LOE expected price level of branded product^b^	54	54	54	54	54	54
Post-LOE expected price level of generics/biosimilars^b^	54	54	54	54	54	54
**Retail**						
Market share of generics/biosimilar (%)	60	70	80	90	90	90
Market growth/expansion post-LOE (%)	0	0	0	0	0	0
Post-LOE expected price level of branded product^b^	65	41	41	41	41	41
Post-LOE expected price level of generics/biosimilars^b^	65	41	41	41	41	41
**Sweden**						
**Hospital**						
Market share of generics/biosimilar (%)	60	100	100	100	100	100
Market growth/expansion post-LOE (%)	0	0	0	0	0	0
Post-LOE expected price level of branded product^b^	60	50	30	30	30	30
Post-LOE expected price level of generics/biosimilars^b^	60	50	30	30	30	30
**Retail**						
Market share of generics/biosimilar (%)	60	100	100	100	100	100
Market growth/expansion post-LOE (%)	0	0	0	0	0	0
Post-LOE expected price level of branded product^b^	100	35	35	35	35	35
Post-LOE expected price level of generics/biosimilars^b^	20	10	10	10	10	10

^a^ Inputs were based on local settings with regards to expected changes in the market dynamics post-LOE, with different inputs used for hospital and retail sectors, as pharmaceuticals are funded through different routes across these channels, with different procurement processes and regulations on pricing and uptake post-LOE.^19–23^

^b^ Percentage of the branded product’s price pre-LOE.

^c^ For the Netherlands, different inputs were used for biological products and small molecules, as policies and uptake of generics and biosimilars differ. Within this, the assumptions for hospital and retail pharmaceuticals are the same.

### Sensitivity Analysis

One-way sensitivity analysis of the average budget made available annually due to LOE during 2020-2024 was conducted for both the hospital and retail cases.

## RESULTS

### Greece

**Figure 1.**
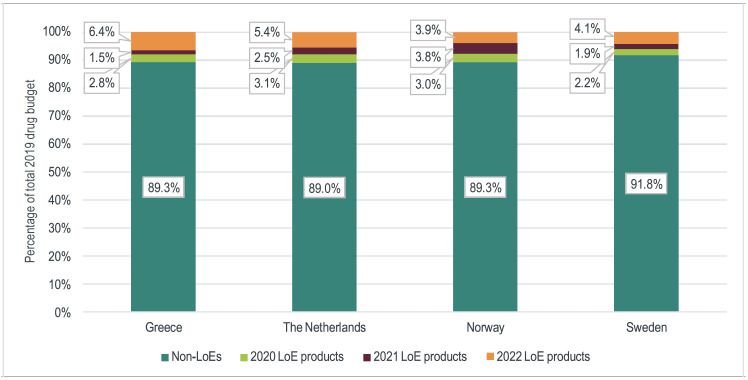
2019 Sales Value of LOE Products as a Share of the Total Drug Budget

In Greece, the drug budget in 2019 was €2424 million, representing 26.2% of the healthcare budget.^25^ The combined annual sales of the 166 products identified as losing exclusivity from January 2020 to December 2022 was €260 million in 2019, representing 10.8% of the total drug budget **(Figure 1)**. A total of €68 million (2.8% of the 2019 drug budget) was spent on products losing exclusivity in 2020, €35 million (1.5%) on products losing exclusivity in 2021, and €156 million (6.4%) on products losing exclusivity in 2022 **(Figure 1)**. While more products faced LOE in the hospital setting (n=90) compared with the retail setting (n=76), hospital products represented a smaller proportion of the total annual drug budget (2.9% vs 7.8%) **(Table 2)**.

**Table 2.** Number of LOE Products, Sales Value of LOE Products, and Pre-LOE Sales Value of LOE Products as a Share of the Annual Drug Budget

	**No. of LOE Products**	**2019 Pre-LOE Sales Value** (€)	**Sales Value of LOE Products as Share of Total Annual Drug Budget (%)**
**Greece**			
Hospital	90	71 171 730^a^	2.9
Retail	76	188 444 076^a^	7.8
Total	166	259 615 806^a^	10.7
**The Netherlands**			
Hospital	40^b^	384 123 983	6.7
Retail	182^b^	244 103 291	4.3
Total	222	628 227 274	11.0
**Norway**			
Hospital	83	119 652 505	6.0
Retail	62	93 367 755	4.7
Total	145	213 020 260	10.7
**Sweden**			
Hospital	28	59 358 267	1.3
Retail	65	307 121 237	6.9
Total	93	366 479 504	8.2

^a^ For Greece, sales values for retail products were from 2019 and for hospital products were from 2018 consumption data.

^b^ Includes products used in both hospital and retail sectors.

**Figure 2.**
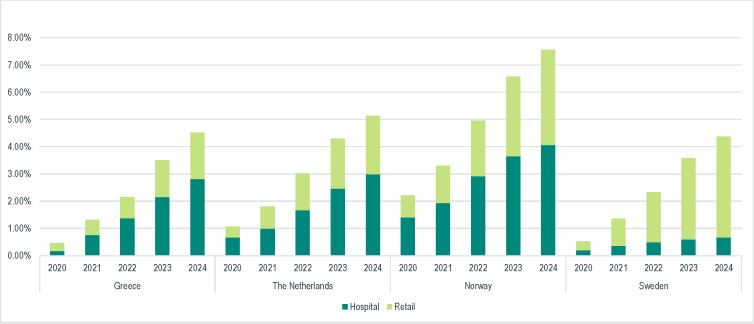
Average Budget Realized as a Share of Total Annual Drug Budget (2020-2024)

The total forecasted LOE-related budget made available for potential reallocation for 2020-2024 was €218 million, increasing from €3 million (0.47% of total drug budget) to €87 million (4.53% of total drug budget) **(Figure 2; Table 3).** The average budget available per year from 2020-2024 represented a 1.8% share of the total annual drug budget, including a 1% annual increase in drug budget from 2019-2024. The budget made available for hospital and retail sectors for 2020-2024 is presented in Table 3.

**Table 3.** Estimated Budget Realized as a Result of LOE and as a Share of Annual Drug Budget, 2020-2024

	**Estimated Budget Made Available, by Year**					
	**2020**	**2021**	**2022**	**2023**	**2024**	**2020-2024 Total**
**Greece**						
Hospital (€)	416 983	4 863 072	10 884 012	19 519 502	24 902 615	60 586 185
Retail (€)	2 868 069	13 665 183	22 292 164	56 886 371	61 947 967	157 659 754
Total budget made available per year (€)	3 285 052	18 528 255	33 176 176	76 405 873	86 850 582	218 245 939
Budget made available as share of total annual drug budget (%)	0.47	1.31	2.15	3.50	4.53	—
**The Netherlands**						
Hospital (€)	19 438 120	65 553 495	152 718 052	254 214 634	277 193 217	769 117 519
Retail (€)	11 010 390	58 804 137	122 789 521	173 272 753	183 688 122	549 564 924
Total budget made available per year (€)	30 448 511	124 357 632	275 507 573	427 487 387	460 881 339	1 318 682 442
Budget made available as share of total annual drug budget (%)	1.07	1.81	3.02	4.30	5.14	—
**Norway**						
Hospital (€)	7 048 626	22 120 315	43 903 977	55 040 151	55 040 151	183 153 221
Retail (€)	4 001 176	16 460 761	30 490 177	51 118 828	55 086 976	157 157 917
Total budget made available per year (€)	11 049 802	38 581 076	74 394 154	106 158 979	110 127 127	340 311 138
Budget made available as share of total annual drug budget (%)	2.21	3.30	4.96	6.58	7.56	—
**Sweden**						
Hospital (€)	4 582 271	19 789 264	31 070 002	38 742 186	41 550 787	135 734 510
Retail (€)	7 210 436	59 681 922	137 361 914	259 726 241	276 409 114	740 389 628
Total budget made available per year (€)	11 792 707	79 471 186	168 431 916	298 468 427	317 959 901	876 124 138
Budget made available as share of total annual drug budget (%)	0.53	1.37	2.33	3.58	4.37	—

LOE for products in the alimentary tract and metabolism ATC group had the greatest contribution to the budget made available for reallocation, followed by antineoplastic and immunomodulating agents, with a high number of products facing LOE in these groups. The full breakdown of LOE products and budget made available by ATC group over time is presented in **Supplementary Tables 2 and 6.**

### The Netherlands

In the Netherlands, the drug budget in 2019 was €5700 million, representing 7.3% of the healthcare budget.^25^ The combined annual sales of the 222 products identified losing exclusivity from January 2020 to December 2022 was €628 million in 2019, representing 11.0% of the total drug budget (Figure 1). A total of €177 million (3.1% of the 2019 drug budget) was spent on products losing exclusivity in 2020, €145 million (2.5%) on products losing exclusivity in 2021, and €306 million (5.4%) on products that will lose exclusivity in 2022 **(Figure 1).** More products with LOE were identified in the retail setting (n=182) than in the hospital setting (n=40), while products facing LOE in the hospital channel represented a greater share of the total annual drug budget (6.7% in hospital channel, 4.3% in retail channel) **(Table 2)**.

The LOE-related budget made available for 2020-2024 was €1319 million, increasing from €30 million (1.07% of total drug budget) to €461 million (5.14% of total drug budget) **(Figure 2; Table 3).** The average budget made available per year from 2020-2024 represented a 4.6% share of the total annual drug budget assuming constant drug budget over the model time horizon. The budget made available for hospital and retail channels for 2020-2024 is presented in Table 3.

The ATC group contributing most to the budget availability was antineoplastic and immunomodulating agents, followed by the nervous system group. These were among the groups with the highest number of products facing LOE and resulted in a high budget made available per LOE. While products in the musculoskeletal group contributed a low total budget availability, the per-product impact was higher than the other groups. The full breakdown of number of LOE products and the budget made available by ATC group over time is presented in **Supplementary Tables 3 and 7**.

### Norway

In Norway, the drug budget in 2019 was €2000 million, representing 7.3% of the healthcare budget.^25^ The combined annual sales of the 145 products identified as losing exclusivity from January 2020 to December 2022 was €213 million in 2019, representing 10.7% of the total drug budget **(Figure 1)**. A total of €60 million (3.0% of the 2019 drug budget) was spent on products losing exclusivity in 2020, €76 million (3.8%) on products losing exclusivity in 2021, and €77 million (3.9%) on products that will lose exclusivity in 2022 **(Figure 1)**. In Norway, 83 products with LOE were identified in the hospital channel, representing 6.0% of the total annual drug budget compared with 62 in the retail channel (4.7% of the annual drug budget) **(Table 2)**.

The total forecasted LOE-related budget made available for 2020-2024 was €340 311 138, increasing from €11 million (2.21% of total drug budget) to €110 million (7.56% of total drug budget) **(Figure 2; Table 3)**. The average budget made available per year from 2020-2024 represented a 3.4% share of the total annual drug budget in 2019. The budget made available for hospital and retail channels for 2020-2024 is presented in Table 3.

LOE for products from the ATC groups antineoplastic and immunomodulating agents, nervous system, and alimentary tract and metabolism contributed most to the estimated budget availability, while antiparasitic products, insecticides and repellents, and systemic hormonal preparations (excluding sex hormones and insulins) had little impact on the budget availability. Products in the musculoskeletal group had a high per-product impact on the budget made available. The full breakdown of the number of LOE products and budget made available by ATC group over time is presented in **Supplementary Tables 4 and 8**.

### Sweden

In Sweden, the drug budget in 2019 was €4454 million, representing 9.8% of the healthcare budget.^25^ The combined annual sales of the 93 products identified losing exclusivity from January 2020 to December 2022 was €366 million in 2019, representing 8.2% of the total drug budget **(Figure 1)**. A total of €97 million (2.2% of the 2019 drug budget) was spent on products losing exclusivity in 2020, €87 million (1.9%) on products losing exclusivity in 2021, and €182 million (4.1%) on products that will lose exclusivity in 2022 **(Figure 1)**. In Sweden, more than twice as many products with LOE were identified in the retail channel (n=65) compared with the hospital channel (n=28), corresponding to a sales value of 6.9% and 1.3% of the total annual drug budget, respectively **(Table 2)**.

The total forecasted LOE-related budget made available for 2020-2024 was €876 million, increasing from €12 million (0.53% of total drug budget) to €318 million (4.37% of total drug budget) **(Figure 2; Table 3)**. The average budget made available per year from 2020-2024 represented a 3.9% share of the total annual drug budget assuming constant drug budget over the model time horizon. The budget made available for hospital and retail channels for 2020-2024 is presented in Table 3.

The group contributing most to budget availability was antineoplastic and immunomodulating agents, followed by the nervous system group. These groups had the highest number of LOE products and represent groups with a higher per-product contribution to budget availability. LOE in the blood and blood-forming organs group had a low impact on the budget made available, as well as a low number of LOE products. Products in the musculoskeletal group had the highest per-product impact on the budget made available. The full breakdown of the number of LOE products and budget made available by ATC group over time is presented in **Supplementary Tables 5 and 9**.

### Sensitivity Analysis

Table 4 presents the one-way sensitivity analysis on the total budget made available for reallocation for each country. Although the same driving factors existed in each country, the magnitude of the impact of each variable was different between countries. The hospital sector showed a large variation in the impact of changing the price level of branded drugs or generics/biosimilars on the total budget made available across the countries. This impact ranged from a 0.8% increase in Sweden to a 21.7% increase in Norway when the price level was decreased by 10%. Meanwhile, the impact of increasing generic uptake by an additional 10% relative to the base case ranged from a 2.7% increase in the budget made available in the Netherlands, to 28.8% in Greece, while a delay in generic/biosimilar entry by 2 cycles (equating to 1 year) decreased the budget made available by as much as -30.1% in Norway and had a huge impact in Sweden. Similar results were seen in the retail sector, although in Greece, decreasing generic price levels was the only variable to impact budget availability.

**Table 4.** One-Way Sensitivity Analysis on the Average Budget Made Available due to LOE During 2020-2024

**Scenario**	**Estimated Average Budget Made Available per Year During 2020-2024 (€)**	**Average Budget Made Available (% of Average Annual Drug Budget During 2020-2024)**	**% Change Relative to Base Case**
**Greece**			
**Hospital base case**	**12 117 237**	**0.5**	—
**Change from base case**			
Price level of branded product (-10% of base case) starting in cycle 2	14 486 246	0.60	19.6
Price level of branded product (+10% of base case)	—	—	—
Price levels of generics/biosimilars (-10% of base case)	14 639 458	0.60	20.8
Price levels of generics/biosimilars (+10% of base case)	—	—	—
Increased generics/biosimilars uptake by additional 10% of base case for 3 cycles	15 606 987	0.64	28.8
Delay of generics/biosimilars entrance by 2 cycles	9 406 386	0.39	-22.4
**Retail base case**	**31 531 951**	**1.30**	—
**Change from base case**			
Price level of branded product (-10% of base case) starting in cycle 2	31 531 951	1.30	0
Price level of branded product (+10% of base case)	—	—	—
Price levels of generics/biosimilars (-10% of base case)	37 746 891	1.56	19.7
Price levels of generics/biosimilars (+10% of base case)	—	—	—
Increased generics/biosimilars uptake by additional 10 of base case for 3 cycles	31 531 951	1.30	0
Delay of generics/biosimilars entrance by 2 cycles	31 531 951	1.30	0
**The Netherlands**			
**Hospital base case**	**192 279 380**	**3.37**	—
**Change from base case**			
Price level of branded product (-10% of base case)	202 077 027	3.55	5.1
Price level of branded product (+10% of base case)	182 481 733	3.20	-5.1
Price levels of generics/biosimilars (-10% of base case)	209 885 016	3.68	9.2
Price levels of generics/biosimilars (+10% of base case)	172 294 991	3.02	-10.4
Increased generics/biosimilars uptake by additional 10% of base case for 3 cycles	197 525 697	3.47	2.7
Delay of generics/biosimilars entrance by 2 cycles	181 806 923	3.19	-5.4
**Retail base case**	**137 391 231**	**2.41**	—
**Change from base case**			
Price level of branded product (-10% of base case)	143 759 494	2.52	4.6
Price level of branded product (+10% of base case)	131 022 968	2.30	-4.6
Price levels of generics/biosimilars (-10% of base case)	145 356 908	2.55	5.8
Price levels of generics/biosimilars (+10% of base case)	124 123 342	2.18	-9.7
Increased generics/biosimilars uptake by additional 10% of base case for 3 cycles	141 280 441	2.48	2.8
Delay of generics/biosimilars entrance by 2 cycles	127 741 211	2.24	-7.0
**Norway**			
**Hospital base case**	**36 630 644**	**1.83**	—
**Change from base case**			
Price levels of branded and generics/biosimilars (-10% of base case)	44 593 828	2.23	21.7
Price levels of branded and generics/biosimilars (+10% of base case)	28 667 461	1.43	-21.7
Increased generics/biosimilars uptake by additional 10% of base case for 3 cycles	—	—	—
Delay of generics/biosimilars entrance by 2 cycles – branded price remains at max price for 2 cycles	25 622 614	1.28	-30.1
**Retail base case**	**31 431 583**	**1.57**	—
**Change from base case**			
Price levels of branded and generics/biosimilars (-10% of base case)	37 138 772	1.86	18.2
Price levels of branded and generics/biosimilars (+10% of base case)	25 724 395	1.29	-15.4
Increased generics/biosimilars uptake by additional 10% of base case for 3 cycles	—	—	—
Delay of generics/biosimilars entrance by 2 cycles – branded price remains at max price for 2 cycles	20 414 188	1.02	-42.8
**Sweden**			
**Hospital base case**	**31 301 981**	**0.70**	—
**Change from base case**			
Price level of branded product (-10% of base case)	31 539 414	0.71	0.8
Price level of branded product (+10% of base case)	31 064 548	0.70	-0.8
Price levels of generics/biosimilars (-10% of base case)	35 960 247	0.81	14.9
Price levels of generics/biosimilars (+10% of base case)	26 643 715	0.60	-14.9
Increased generics/biosimilars uptake by additional 10 of base case for 3 cycles	39 920 917	0.90	27.5
Delay of generics/biosimilars entrance by 2 cycles	18 836 744	0.47	-60.2
**Retail base case**	**175 718 837**	**3.95**	—
**Change from base case**			
Price level of branded product (-10% of base case)	176 947 322	3.97	0.7
Price level of branded product (+10% of base case)	175 718 837	3.95	0
Price levels of generics/biosimilars (-10% of base case)	193 659 299	4.35	10.2
Price levels of generics/biosimilars (+10% of base case)	155 989 774	3.50	-11.2
Increased generics/biosimilars uptake by additional 10% of base case for 3 cycles	222 722 033	5.00	26.7
Delay of generics/biosimilars entrance by 2 cycles	88 570 244	2.21	-50.4

## DISCUSSION

Overall, the results presented here demonstrate that LOE for branded products has the potential to generate a considerable healthcare budget available for reallocation up to 2024, across all 4 countries included in the analysis. Importantly, differences between countries may be explained by the differing inputs of expected changes in the market dynamics post-LOE; therefore, any direct comparisons between countries should be made with caution. All 4 countries have largely publicly-funded systems and report a similar level of spending on health care (7.8%-10.9% of GDP), and similar level of the healthcare budget being spent on pharmaceuticals.^25–27^ However, there are differences in the management of this budget and post-LOE restrictions, reflected in the different levels of budget availability estimated; these range from an average of 1.8% of the drug budget in Greece to 4.6% of the drug budget in the Netherlands.

All 4 countries showed year-on-year increases in the annual budget generated for reallocation, including in 2023 and 2024, when further LOE was not accounted for. The increase in the budget made available began to plateau in 2024 as LOE beyond 2020-2022 was not accounted for in this analysis. While data for the Netherlands, Norway, and Sweden showed a linear upward trend in the budget made available each year from 2020-2023, Greece had a steeper increase between 2022 and 2023, likely driven by the higher number of products losing exclusivity in 2022.

The countries analyzed each have different policies around pricing of branded products and generics/biosimilars following LOE. Of note, Norway has a well-defined structure for post-LOE pricing through the stepped price system.^19^ Within this system, which applies to all retail products, the price of the branded product is reduced stepwise at generic entry, after 6 months, and after 18 months, with the price cuts defined by product sales.^19^ Within each year, the estimated budget made available in Norway represented the highest share of the total drug budget from the countries included, even though only 2 of the 3 price cuts were considered in this analysis, highlighting the potential for greater budget gains post-LOE. Nevertheless, in the absence of firm policies for price reductions post-LOE, high budget can still be made available due to increased competition, as exemplified by the hospital sector in Sweden. It is noted that Sweden also has a product-of-the-month (Svenska Periodens Vara) system for pharmacy-dispensed pharmaceuticals to reduce prices post-LOE; this also contributes to the budget made available.^28^

In the Netherlands, Norway, and Sweden, antineoplastic and immunomodulating agents were the main drivers of the budget made available, with this group also having a large contribution in Greece. Products in the alimentary tract and metabolism group generally had a high contribution to the budget made available, and drugs in the nervous system group had a high contribution in all countries except for Greece. These trends can largely be explained by a higher sales value of products losing exclusivity in these groups during the time horizon of the analysis. In all countries except Greece, while LOE in the musculoskeletal group did not play a large role overall in generating budget for reallocation, the per-product share of funds freed up was high.

The drivers of budget made available for reallocation in terms of sales channels differed across the countries. In Greece, LOE in the retail channel was the main driver of additional budget availability, despite a higher number of products facing LOE in the hospital setting. This may be explained by the high use of products from the alimentary tract and metabolism ATC group in the retail setting. In Sweden, the majority of products facing LOE were in retail, which is reflected in this sector being the main driver of estimates of the budget made available in these analyses. In the Netherlands and Norway, estimated budget made available was driven by LOE in the hospital channel, where products in the ATC group of antineoplastic and immunomodulating agents gained most use. It is worth noting that ATC groups covered within the hospital and retail sectors may vary by country, which can have a significant impact on the budget realized in each sector–a key consideration given that hospital and retail budgets are managed separately in these countries.

For each country, the analysis demonstrates the areas in which higher budget can be realized, including which sales channels and which ATC groups would enable the highest savings as a result of LOE. The ART model can be used further to estimate the future budget availability in other European markets.

It is important to consider that a conservative approach was adopted for this model, which led to a slight underestimation of the budget made available for reallocation. For example, for Greece, the IQVIA sales data were based on wholesale prices (excluding value-added tax and private pharmacies’ profit margin). Also, public purchases considered retail prices excluding patient copayments. In general, generic medicines enter the market at 65% of the pre-LOE price of the branded medicines, with price reassessment occurring once per year at different levels for generics versus branded products. To account for this, the base case of the analysis included a 7% annual price decrease (the maximum annual price decrease for off-patent medicines) for all medicines in both sectors.

The sensitivity analysis showed varying dependency on different inputs by country. In Greece, the budget made available in the hospital setting was sensitive to increasing generic/biosimilar uptake, while in the retail setting, mainly changing the price levels of generics had an impact on the budget made available. In the Netherlands, the budget made available was most sensitive to price changes of generics/biosimilars in both hospital and retail channels, with a relatively high impact in the retail channel when delaying generic/biosimilar entry by 2 cycles. For Norway, delaying generic/biosimilar entry by 2 cycles had the greatest impact on budget made available in both hospital and retail settings, although there were sensitivities to other factors. Notably, in Sweden, delaying generic/biosimilar entry had the greatest impact on the budget made available in the hospital channel. In both hospital and retail channels, the budget made available in Sweden was most sensitive to delayed generic/biosimilar entry.

Of the countries included in this analysis, currently none have proactive processes to evaluate the potential budget made available from LOE and manage the reallocation of these funds. The introduction of policies to ensure appropriate reallocation of funds, alongside policies to manage the market dynamics post-LOE, can help to support an efficient and structured approach to budgeting.

Importantly, the IQVIA MIDAS® dataset does not provide estimates for the LOE date for all products, with the data gap varying between 57% and 75%, depending on the country. The impact of these data gaps is expected to be limited, since products for which the LOE dates are missing account for only 18% to 30% of the total sales value. The results of this study therefore provide a conservative estimate of the budget made available, as there may exist additional LOE products that were not included in these analyses. Furthermore, the IQVIA sales values used within the ART model relate to the products’ list prices and do not consider confidential net price agreements; therefore, they may not reflect the actual prices paid. In addition, the analysis does not consider the class effect of LOE when estimating the budget available for reallocation. When the LOE product is the first in its product class to lose exclusivity, the market shares and prices of other products in the same product class may decrease, leading to an even larger decrease in total sales values and thus a higher budget made available when product class is taken into account.

This approach has a number of other limitations. First, the accuracy in the estimated budget made available will depend on the accuracy of the country-specific model inputs, which are largely based on expectations informed by observation of historical changes in market dynamics post-LOE. These inputs include the market share of branded products relative to generics/biosimilars, the market expansion post-LOE, and the price decrease of the branded drug and price of generics/biosimilars. Market expansion was set to 0% in the analysis because, in these markets, expansion post-LOE was not expected for mature products. If market expansion were to occur, market share would most likely come from non-LOE drugs (if any exist within the class in question), potentially leading to further budget available for reallocation due to the class effect post-LOE. While in some countries the price levels post-LOE are mandated by local legislation, in others, the decreases have been assumed based on experience. Second, the baseline annual sales values for LOE products are not extrapolated to the LOE timepoint since this would add additional uncertainty in the calculations. Instead, the calculations use the known most recent information on the MAT for each product and assume that the annual sales values will stay unchanged until the LOE timepoint. As a result, the budget estimates are more accurate for the LOE products 1-3 years in the future than those further out.

## CONCLUSION

This analysis may support more efficient management of budgets to ensure availability of treatments to promote increased overall health and well-being. Budget predictability is essential for decision makers in health care. Estimation of future budget made available adds to the discussion on resource allocation and may help inform policy changes.

The ART model scenario analyses in Greece, the Netherlands, Norway, and Sweden showed that products losing exclusivity between 2020 and 2022 can contribute to significant budget availability. Strategies are needed to ensure future budgets optimize reallocation to therapy areas benefiting from new innovations. Although patents and other policies enabling product exclusivity are paramount to drive innovation and advancement in drug development, when this period of exclusivity ends there is an opportunity to reallocate funds. Introduction of policies to forecast and manage budget availability and potential reallocation of these funds should be considered.

Future research should look to examine the whether the ART model can be used to establish the budget available for reallocation following LOE in larger healthcare systems such as France, Germany and the UK. In addition, it would be beneficial to understand how the principles behind the ART model could be applied to the US market where the federal and state governments, and private healthcare insurers all reallocate budgets differently following LOE.

### Financial Disclosure

Six of the seven authors of this work were employees of MSD at the time of the research. The remaining author received consultancy fees for working on this project together with MSD employees.

### Conflict of Interest Disclosure

None reported.

### Additional Disclosure

The model upon which the manuscript is based was presented at the ISPOR Milan Conference 2020 as an abstract with interim findings. The poster’s abstract was published in: Toghanian S, Papageorgiou M, Kittelsen K, Dolk C, Hultstrand M, Salomonsson S. Estimating future budget headroom as a result of expiration of exclusivity for pharmaceuticals using the Affordability by ReallocaTing Funds (ART) MODEL [Abstract]. *Value Health.* 2020; 23(suppl 2):S660. https://doi.org/10.1016/j.jval.2020.08.1554

## Supplementary Material

Supplementary Online Material
