# Supplementary material for: Estimating Potential for Drug Budget Reallocation Following Expiration of Exclusivity of Pharmaceutical Products
Source: J Health Econ Outcomes Res. 2022 Feb 3;9(1):20–30. doi: 10.36469/jheor.2022.29624 (PMC8813194; doi:10.36469/jheor.2022.29624)
Supplement: Supplementary Online Material [file jheor_2022_9_1_29624_81203.pdf]

Journal of Health Economics and Outcomes Research

Online Supplementary Material

Estimating Potential for Drug Budget Reallocation Following Expiration of Exclusivity of Pharmaceutical Products. *JHEOR*. 2022;9(1):20-30. doi:10.36469/jheor.2022.29624

Figure S1. ART Model Schematic

Table S1. ATC Classification

Table S2. Number of LOE Products, Sales Value of LOE Products, and Pre-LOE Sales Value of LOE Products as a Share of Annual Drug Budget, by ATC Group—Greece

Table S3. Number of LOE Products, Sales Value of LOE Products, and Pre-LOE Sales Value of LOE Products as a Share of Annual Drug Budget by ATC Group—The Netherlands

Table S4. Number of LOE Products, Sales Value of LOE Products, and Pre-LOE Sales Value of LOE Products as a Share of Annual Drug Budget by ATC Group—Norway

Table S5. Number of LOE Products, Sales Value of LOE Products, and Pre-LOE Sales Value of LOE Products as a Share of Annual Drug budget by ATC Group—Sweden

Table S6. Estimated Budget Headroom Realized as a Result of LOE and Budget Headroom as a Share of Annual Drug Budget by ATC Group, 2020-2024—Greece

Table S7. Estimated Budget Headroom Realized as a Result of LOE and Budget Headroom as a Share of Annual Drug Budget by ATC Group, 2020-2024—The Netherlands

Table S8. Estimated Budget Headroom Realized as a Result of LOE and Budget Headroom as a Share of Annual Drug Budget by ATC Group, 2020-2024—Norway

Table S9. Estimated Budget Headroom Realized as a Result of LOE and Budget Headroom as a Share of Annual Drug Budget by ATC Group, 2020-2024—Sweden

This supplementary material has been provided by the authors to give readers additional information about their work.

This is an open-access article distributed under the terms of the Creative Commons Attribution 4.0 International License (CCBY-4.0). View this license's legal deed at http://creativecommons.org/licenses/by/4.0 and legal code at http://creativecommons.org/licenses/by/4.0/legalcode for more information.

Figure S1. ART Model Schematic

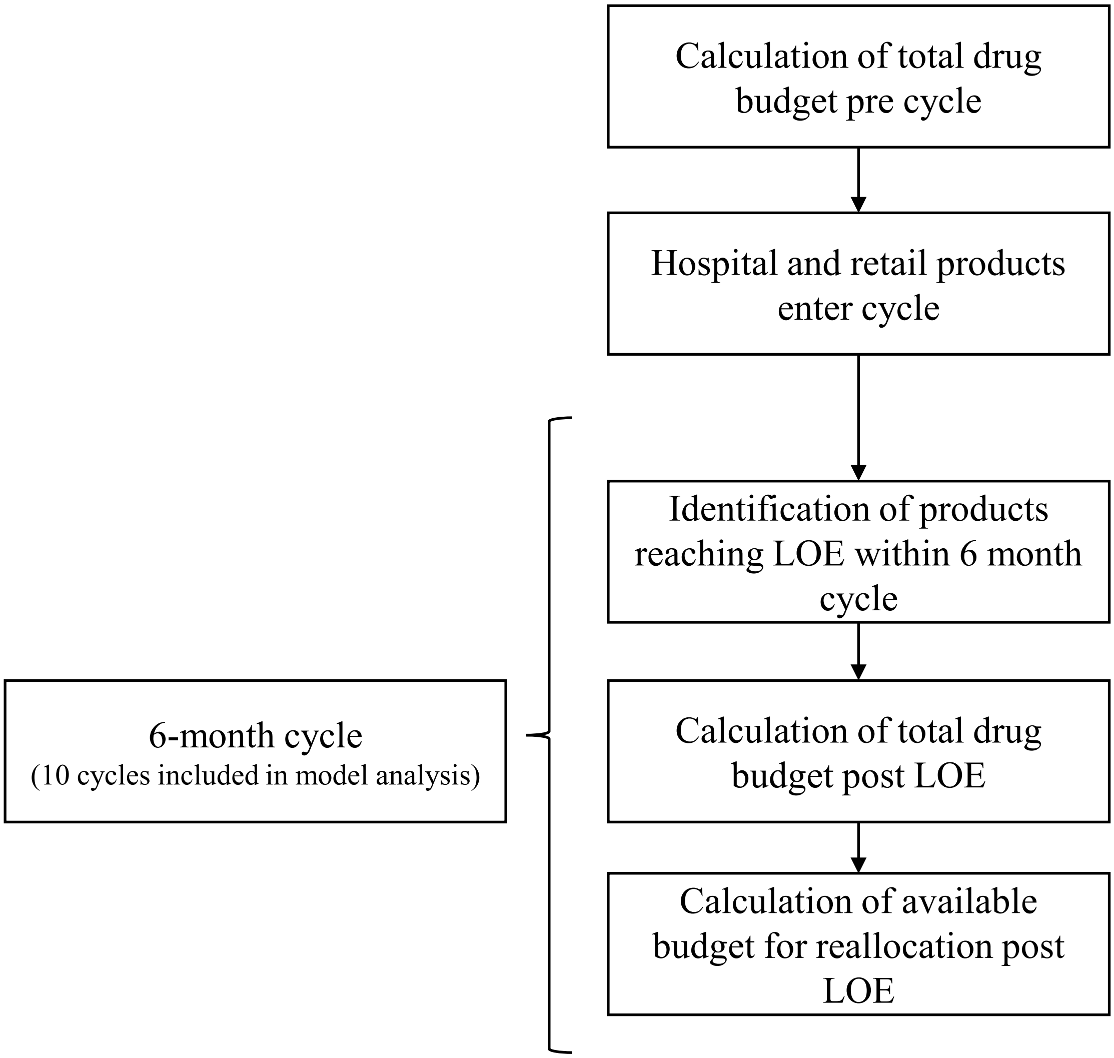

Table S1. ATC Classification[a]

ATC Group	Examples
Alimentary tract and metabolism	Stomatological preparations; drugs for acid-related disorders; drugs for functional gastrointestinal disorders; antiemetics and antinauseants; bile and liver therapy; drugs for constipation; antidiarrheals, intestinal anti-inflammatory/anti-infective agents; anti-obesity preparations, excluding diet products; digestives including enzymes; drugs used in diabetes; vitamins; mineral supplements; tonics; anabolic agents for systemic use; appetite stimulants
Blood and blood-forming organs	Antithrombotic agents; antihemorrhagics; anti-anemic preparations; blood substitutions and perfusion solutions
Dermatologicals	Antifungals for dermatological use; emollients and protectives; preparations for treatment of wounds and ulcers; cicatrizants; enzymes; antipruritics (eg, antihistamines, anesthetics, etc.) antipsoriatics; antibiotics and chemotherapeutics for dermatological use; corticosteroids, dermatological preparations; antiseptics and disinfectants; medicated dressings; anti-acne preparations
Genitourinary system and sex hormones	Gynecological anti-infectives and antiseptics; other gynecologicals; sex hormones and modulators of the genital system; progestogens; urologicals
Antineoplastic and immunomodulating agents	Antineoplastic agents (eg, alkylating agents, antimetabolites, cytotoxic antibiotics); endocrine therapy; immunostimulants; immunosuppressants
Musculoskeletal system	Anti-inflammatory and antirheumatic products; topical products for joint and muscular pain; muscle relaxants; anti-gout preparations, drugs for treatment of bone diseases
Nervous system	Anesthetics; analgesics; antiepileptics; anti-Parkinson drugs; psycholeptics; psychoanaleptics; other nervous system drugs; drugs used in addictive disorders
Respiratory system	Nasal preparations; throat preparations; drugs for obstructive airway diseases; cough and cold preparations; antihistamines for systemic use; other respiratory system products
Various	Allergens; diagnostic agents; general nutrients; therapeutic radiopharmaceuticals; all other nontherapeutic products
Cardiovascular system	Cardiac therapy; antihypertensives; diuretics; peripheral vasodilators; vasoprotectives; beta-blocking agents; calcium channel blockers; agents acting on the renin-angiotensin system; lipid-modifying agents
Anti-infectives for systemic use	Antibacterials for systemic use; antimycotics for systemic use; antimycobacterials; antivirals for systemic use; immune sera and immunoglobulins; vaccines
Sensory organs	Ophthalmologicals; otologicals; ophthalmological and otological preparations
Systemic hormonal preparations, excluding sex hormones and insulins	Pituitary and hypothalamic hormones and analogues; corticosteroids for systemic use; thyroid therapy; pancreatic hormones; calcium homeostasis
Antiparasitic products, insecticides and repellents	Antiprotozoals; anthelmintics; ectoparasiticides, including scabicides; insecticides and repellents

Abbreviation: ATC, Anatomical Therapeutic Chemical.

[a] ATC level 4 classification was used in the ART model calculations to ensure the necessary granularity, with the results aggregated by ATC level 1 when presented.

Source: WHO Collaborating Centre for Drug Statistics Methodology. International language for drug utilization research. Accessed October 2020. https://www.whocc.no/

Table S2. Number of LOE Products, Sales Value of LOE Products, and Pre-LOE Sales Value of LOE Products as a Share of Annual Drug Budget, by ATC Group—Greece

	No. of LOE Products	2019 Sales Value Pre-LOE (€)	Sales Value of LOE Products as Share of Total Annual Drug Budget (%)
Alimentary tract and metabolism	24	92 492 631	3.82
Blood and blood-forming organs	6	6 593 717	0.27
Dermatologicals	8	5 615 944	0.23
Genitourinary system and sex hormones	14	15 498 810	0.64
Antineoplastic and immunomodulating agents	36	33 280 394	1.37
Musculoskeletal system	5	1 118 219	0.05
Nervous system	21	10 639 196	0.44
Respiratory system	13	29 244 161	1.21
Various	5	8 798 504	0.36
Cardiovascular system	11	19 814 552	0.82
Anti-infectives for systemic use	10	10 901 706	0.45
Sensory organs	11	23 120 288	0.95
Systemic hormonal preparations excluding sex hormones and insulins	2	2 497 685	0.10
Antiparasitic products, insecticides, and repellents	0	0	0

Abbreviation: ATC, Anatomical Therapeutic Chemical.

Source: MIDAS® data, IQVIA, NC, USA.

Table S3. Number of LOE Products, Sales Value of LOE Products, and Pre-LOE Sales Value of LOE Products as a Share of Annual Drug Budget by ATC Group—The Netherlands

	No. of LOE Products	2019 Sales Value Pre-LOE (€)	Sales Value of LOE Products as Share of the Total Annual Drug Budget (%)
Alimentary tract and metabolism	50	45 435 907	0.80
Blood and blood-forming organs	1	1779	0.00
Dermatologicals	6	8 242 457	0.14
Genitourinary system and sex hormones	21	17 017 692	0.30
Antineoplastic and immunomodulating agents	42	285 931 902	5.02
Musculoskeletal system	6	52 441 404	0.92
Nervous system	36	93 523 200	1.64
Respiratory system	15	55 051 530	0.97
Various	1	7 519 018	0.13
Cardiovascular system	10	7 471 820	0.13
Anti-infectives for systemic use	13	11 954 460	0.21
Sensory organs	18	31 548 466	0.55
Systemic hormonal preparations excluding sex hormones and insulins	3	12 087 638	0.21
Antiparasitic products, insecticides, and repellents	0	0	0

Abbreviation: ATC, Anatomical Therapeutic Chemical.

Source: MIDAS® data, IQVIA, NC, USA.

Table S4. Number of LOE Products, Sales Value of LOE Products, and Pre-LOE Sales Value of LOE Products as a Share of Annual Drug Budget by ATC Group—Norway

	No. of LOE Products	2019 Sales Value Pre-LOE (€)	Sales Value of LOE Products as Share of the Total Annual Drug Budget (%)
Alimentary tract and metabolism	31	34 348 208	1.72
Blood and blood-forming organs	1	52 342	0.00
Dermatologicals	4	3 388 285	0.17
Genitourinary system and sex hormones	9	8 260 305	0.41
Antineoplastic and immunomodulating agents	21	65 463 436	3.27
Musculoskeletal system	3	9 223 811	0.46
Nervous system	26	58 957 402	2.95
Respiratory system	10	18 803 748	0.94
Various	3	1 837 502	0.09
Cardiovascular system	16	4 447 630	0.22
Anti-infectives for systemic use	9	4 436 656	0.22
Sensory organs	9	3 424 933	0.17
Systemic hormonal preparations, excluding sex hormones and insulins	2	375 620	0.02
Antiparasitic products, insecticides, and repellents	1	382	0.00

Abbreviation: ATC, Anatomical Therapeutic Chemical.
Source: MIDAS® data, IQVIA, NC, USA.

Table S5. Number of LOE Products, Sales Value of LOE Products, and Pre-LOE Sales Value of LOE Products as a Share of Annual Drug Budget by ATC Group—Sweden

	No. of LOE Products	2019 Sales Value Pre-LOE (€)	Sales Value of LOE Products as Share of Total Annual Drug Budget (%)
Alimentary tract and metabolism	17	64 876 089	1.46
Blood and blood-forming organs	1	13 586	0.00
Dermatologicals	3	4 496 281	0.10
Genitourinary system and sex hormones	8	4 428 524	0.10
Antineoplastic and immunomodulating agents	15	116 967 799	2.63
Musculoskeletal system	3	34 426 739	0.77
Nervous system	18	70 357 240	1.58
Respiratory system	5	22 293 107	0.50
Various	2	3 862 330	0.09
Cardiovascular system	5	9 075 494	0.20
Anti-infectives for systemic use	8	7 828 550	0.18
Sensory organs	6	20 069 682	0.45
Systemic hormonal preparations, excluding sex hormones and insulins	2	7 784 083	0.17
Antiparasitic products, insecticides, and repellents	0	0	0

Abbreviation: ATC, Anatomical Therapeutic Chemical.
Source: MIDAS® data, IQVIA, NC, USA.

Table S6. Estimated Budget Headroom Realized as a Result of LOE and Budget Headroom as a Share of Annual Drug Budget by ATC Group, 2020-2024—Greece

	Estimated Budget Headroom, by Year (€)				
	**2020**	**2021**	**2022**	**2023**	**2024**
Alimentary tract and metabolism	240 028	1 575 803	2 551 791	26 450 008	29 346 346
Antineoplastic and immunomodulating agents	378 906	2 735 184	6 083 090	9 483 765	11 712 449
Dermatologicals	606 502	1 368 990	1 519 010	1 931 265	1 964 091
Genitourinary system and sex hormones	1 541 596	3 491 887	4 672 090	5 349 895	5 468 018
Musculoskeletal system	28 192	69 807	133 955	292 975	387 340
Nervous system	157 435	1 276 343	2 818 660	3 392 094	3 775 477
Systemic hormonal preparations excluding sex hormones and insulins	326 921	737 906	818 476	892 576	893 370
Respiratory system	25	354 724	1 963 543	8 452 574	9 386 173
Sensory organs	—	244	718 133	4 823 779	7 245 037
Anti-infectives for systemic use	—	1 011 640	2 370 703	3 413 612	3 935 386
Cardiovascular system	—	4 714 541	6 098 570	6 720 717	7 091 163
Blood and blood-forming organs	289	28 256	1 041 055	1 970 613	2 196 831
Various	5160	1 162 930	2 387 098	3 231 998	3 448 902

Abbreviation: ATC, Anatomical Therapeutic Chemical.

Table S7. Estimated Budget Headroom Realized as a Result of LOE and Budget Headroom as a Share of Annual Drug Budget by ATC Group, 2020-2024—The Netherlands

	Estimated Budget Headroom, by Year (€)				
	**2020**	**2021**	**2022**	**2023**	**2024**
Alimentary tract and metabolism	346 772	7 999 009	10 874 527	29 629 077	33 923 085
Antineoplastic and immunomodulating agents	14 790 373	40 771 080	104 127 633	191 231 274	210 100 187
Blood and blood-forming organs	534	1192	1335	1352	1352
Cardiovascular system	450 915	3 632 932	4 907 090	5 550 098	5 664 778
Dermatologicals	2 321 779	5 185 307	5 804 449	6 183 757	6 254 204
Genitourinary system and sex hormones	2 913 492	6 676 863	10 978 077	12 622 902	12 909 147
Musculoskeletal system	5 998 354	15 595 721	28 011 277	34 696 051	35 788 646
Systemic hormonal preparations, excluding sex hormones and insulins	3 626 291	8 098 717	9 065 728	9 186 605	9 186 605
Nervous system	—	16 441 172	49 437 006	65 994 039	69 046 725
Respiratory system	—	16 547 301	36 891 955	41 289 709	41 839 163
Anti-infectives for systemic use	—	3 408 337	7 281 148	8 799 657	9 062 496
Various	—	—	2 255 705	5 037 742	5 639 264
Sensory organs	—	—	5871.64	17 265 124	21 465 688

Abbreviation: ATC, Anatomical Therapeutic Chemical.

Table S8. Estimated Budget Headroom Realized as a Result of LOE and Budget Headroom as a Share of Annual Drug Budget by ATC Group, 2020-2024—Norway

	**Estimated Budget Headroom by Year (€)**				
	**2020**	**2021**	**2022**	**2023**	**2024**
Alimentary tract and metabolism	698 211	1 789 140	3 352 316	16 678 815	19 905 601
Antineoplastic and immunomodulating agents	2 724 097	6 561 069	20 292 781	30 113 181	30 113 181
Dermatologicals	593 870	1 996 912	1 996 912	1 996 912	1 996 912
Genitourinary system and sex hormones	892 125	2 997 231	3 902 863	4 867 269	4 867 269
Musculoskeletal system	1 073 827	3 897 082	4 736 095	4 736 095	4 736 095
Nervous system	4 224 502	15 062 882	26 921 637	29 523 794	29 523 794
Respiratory system	795 648	3 300 238	8 093 881	10 494 372	10 824 752
Various	46 800	93 599	861 403	1 055 234	1 055 234
Sensory organs	—	302 440	540 546	1 685 855	1 943 662
Cardiovascular system	723	1 878 519	2 564 688	2 598 735	2 598 735
Anti-infectives for systemic use	—	677 362	1 034 094	2 156 389	2 309 564
Systemic hormonal preparations, excluding sex hormones and insulins	—	—	65 879	221 271	221 271
Antiparasitic products, insecticides, and repellents	—	—	176	176	176

Abbreviation: ATC, Anatomical Therapeutic Chemical.

Table S9. Estimated Budget Headroom Realized as a Result of LOE and Budget Headroom as a Share of Annual Drug Budget by ATC Group, 2020-2024—Sweden

	**Estimated Budget Headroom by Year (€)**				
	**2020**	**2021**	**2022**	**2023**	**2024**
Alimentary tract and metabolism	200 864	4 422 363	6 544 626	45 937 222	57 908 280
Antineoplastic and immunomodulating agents	4 476 041	16 139 410	39 035 420	92 452 233	98 230 157
Dermatologicals	582 696	2 299 572	2 528 498	3 618 016	3 932 190
Musculoskeletal system	4 440 773	16 681 249	27 640 756	30 971 465	30 971 465
Nervous system	501 546	24 506 759	50 643 709	62 421 949	62 421 949
Systemic hormonal preparations excluding sex hormones and insulins	1 529 934	5 737 254	6 371 464	6 723 803	6 723 803
Various	60 854	182 562	1 066 923	3 415 243	3 415 243
Anti-infectives for systemic use	—	1 848 889	4 061 334	5 661 350	6 040 213
Blood and blood-forming organs	—	9374	12 227	12 227	12 227
Cardiovascular system	—	4 641 481	6 798 997	6 942 172	6 942 172
Genitourinary system and sex hormones	—	51 868	2 016 827	3 675 386	3 758 143
Respiratory system	—	2 950 404	17 176 912	19 824 294	20 063 797
Sensory organs	—	—	4 534 222	16 813 067	17 540 260

Abbreviation: ATC, Anatomical Therapeutic Chemical.
